# Hepatoprotective Effects of Mushrooms

**DOI:** 10.3390/molecules18077609

**Published:** 2013-07-01

**Authors:** Andréia Assunção Soares, Anacharis Babeto de Sá-Nakanishi, Adelar Bracht, Sandra Maria Gomes da Costa, Eloá Angélica Koehnlein, Cristina Giatti Marques de Souza, Rosane Marina Peralta

**Affiliations:** 1 Department of Biochemistry, State University of Maringá, Maringá 87015-900, Brazil; E-Mails: andasoares7@gmail.com (A.S.S.); anacharis@bol.com.br (A.B.S.-N.); adebracht@uol.com.br (A.B.); cgmsouza@uem.br (C.G.M.S.); 2 Department of Biology, State University of Maringá, Maringá 87015-900, Brazil; E-Mail: sandrafungi51@gmail.com; 3 Department of Nutrition, Federal University of the Southern Frontier, Realeza 85770-000, Brazil; E-Mail: eloa-angelica@hotmail.com

**Keywords:** anthraquinol, anti-oxidant activity, ganoderenic acid, β-glucan, metabolism, natural products, oxidative stress

## Abstract

The particular characteristics of growth and development of mushrooms in nature result in the accumulation of a variety of secondary metabolites such as phenolic compounds, terpenes and steroids and essential cell wall components such as polysaccharides, β-glucans and proteins, several of them with biological activities. The present article outlines and discusses the available information about the protective effects of mushroom extracts against liver damage induced by exogenous compounds. Among mushrooms, *Ganoderma lucidum* is indubitably the most widely studied species. In this review, however, emphasis was given to studies using other mushrooms, especially those presenting efforts of attributing hepatoprotective activities to specific chemical components usually present in the mushroom extracts.

## 1. Introduction

Mushrooms are macrofungi with distinctive basidiomata or ascomata which can be either hypogeous or epigeous, large enough to be seen with the naked eye and to be picked up by hand. The number of different kinds of mushrooms in the earth is estimated to be around 140,000. Estimatively only 10% of the species have already been described and about 2,000 of them are edible. Less than 25 species are largely used as foods, being produced in commercial scale. As food the mushrooms are world-wide appreciated for their taste and flavour and are consumed both in fresh and processed form. They are poor in calories and rich in proteins, fibers, carbohydrates, and important vitamins such as thiamin, riboflavin, ascorbic acid and minerals [1,2,3,4,5]. Studies have demonstrated that the regular consumption of mushrooms or the consumption of isolated bioactive constituents present in mushrooms is beneficial to health. They are usually considered functional foods or nutraceutical products [6,7].

The term *nutraceutical* has been generally used to describe those substances or combination of substances that have considerable potential as dietary supplements and for use in the prevention and treatment of various human diseases without the trouble-some side-effects that frequently accompany treatments involving synthetic drugs. Specifically, a *mushroom nutraceutical* is a refined/partially refined extractive from either the mycelium or the basidioma and ascoma, which is consumed in capsule or tablet form as a dietary supplement (not as a regular food) with potential therapeutic applications [8,9,10].

Medicinal mushrooms have a long history of use in traditional oriental therapies, and fungal metabolites are increasingly being used to treat a wide range of diseases [11,12]. Moreover, edible mushrooms should not be considered simply as food, as some of them have been shown to be rich in bioactive compounds [13]. Mushrooms contain many substances and several of them could have some biological activity. The long list includes polysaccharides, phenolics, proteins (fungal immuno-modulating proteins—FIPs, lectins, glycoproteins and non-glycosylated proteins and peptides), polysaccharide–protein complexes, lipid components (ergosterol), and terpenoids, alkaloids, small peptides and amino acids, nucleotides and nucleosides. This long list represents a great assortment of biological properties which include antioxidant [9,14,15], antitumor/anticancer [16], antimicrobial [13], immunomodulatory [17], anti-inflammatory [18,19], antiatherogenic [20] and hypoglycemic actions [21]. Additionally, hepatoprotective properties have also been reported for mushroom extracts and mushroom-derived molecules [22,23]. The latter property is precisely the main focus of the present report which presents and discusses the current knowledge about the hepatoprotective properties of mushrooms and mushroom-derived molecules. 

## 2. Liver and Biomarkers of Hepatoxicity

The liver is a large, complex organ that is well designed for its central role in carbohydrate, protein and fat metabolism. It is the site where waste products of metabolism, such as ammonia, are detoxified. In conjunction with the spleen it is involved in the destruction of the remnants of the red blood cells and with the recycling of their constituents. It is responsible for synthesizing and secreting bile and for synthesizing lipoproteins and plasma proteins, including clotting factors. It maintains a stable blood glucose level by taking up and storing glucose as glycogen (glycogenesis), breaking it down to glucose when needed (glycogenolysis) and forming glucose from noncarbohydrate sources such as amino acids (gluconeogenesis). The liver also plays an important role in drug elimination and detoxification and liver damage may be caused by many xenobiotics, such as alcohol and many medicines, malnutrition, infection, and anaemia [24,25]. Liver damage is a widespread disease which, in most cases, involves oxidative stress and is characterized by a progressive evolution from steatosis to chronic hepatitis, fibrosis, cirrhosis and hepatocellular carcinoma [26]. 

**Figure 1:**
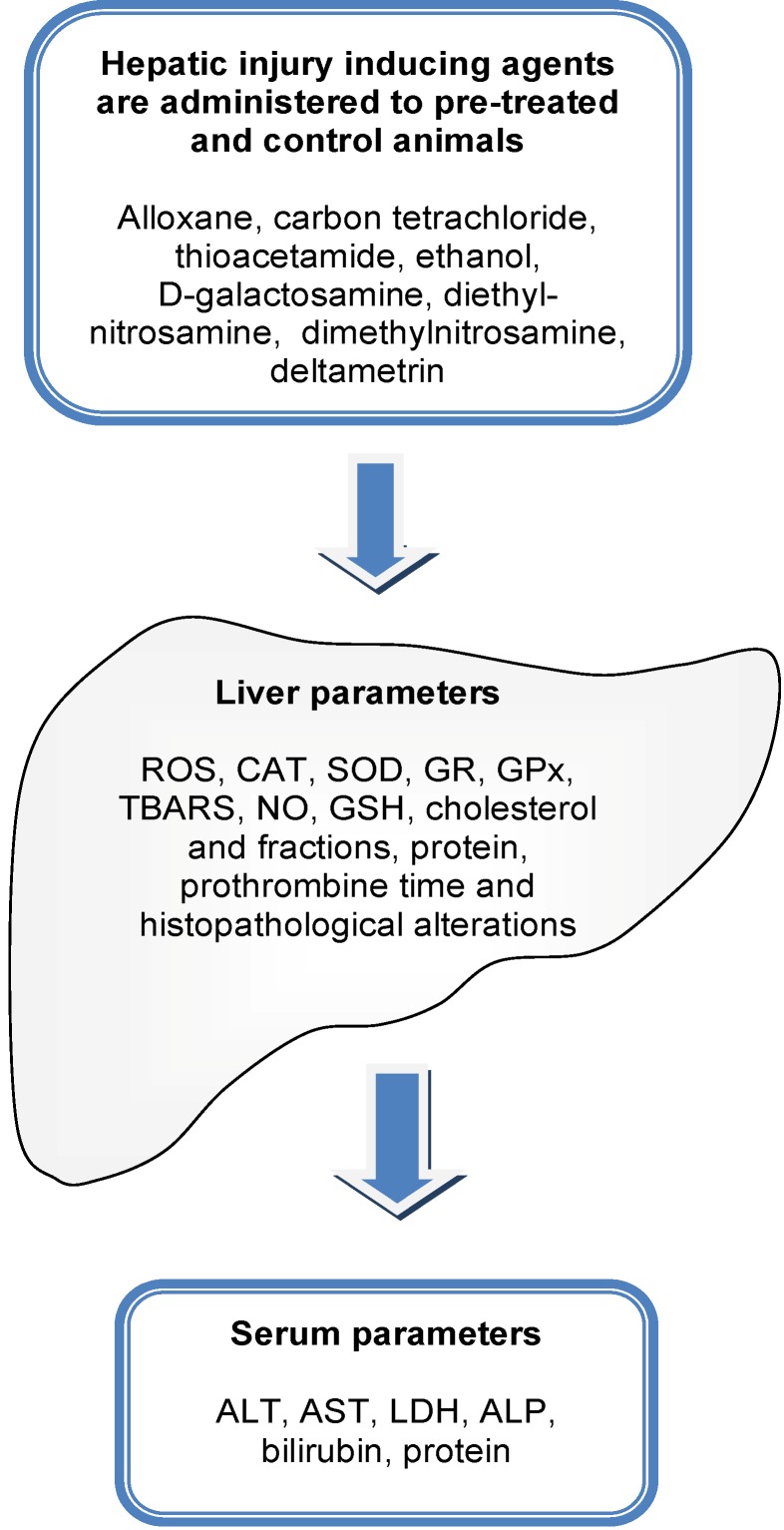
General scheme used for the evaluation of hepato-protective effects of crude or semi-purified extracts and isolated molecules. Animals are treatedfor a specific periodwith a probablehepato-protectiveagent. A lesion is induced by introducing a hepatic damage-inducing agent. Several biomarkers of hepatotoxicity are evaluated in the liver and serum of treated and non-treated animals. TBARS: thiobarbituric acid reactive substances; ROS: reactive oxygen species; CAT: catalase, SOD: superoxide dismutase; GR: glutathione reductase; GPx: gluthatione peroxidase; NO: nitric oxide; GSH: glutathione; ALT: alanine aminotransferase; AST: aspartate aminotransferase; LDH: lactate dehydrogenase; AP: alkaline phosphatase.

The general scheme used to evaluate the hepatoprotective capability of a natural extract or isolated molecule is shown in Figure 1. Hepatotoxicity is defined as an injury to the liver that is associated with an impaired liver function caused by exposure to a drug or another non-infectious agent [27]. Hepatotoxic agents can react with the basic cellular components and consequently induce almost all types of liver lesions. Injury to the liver, whether acute or chronic, eventually results in an increase in serum concentrations of aminotransferases: aspartate aminotransferase (AST) and alanine aminotransferase (ALT). The AST and ALT are enzymes that catalyze the transfer of α-amino groups from aspartate and alanine to the α-keto group of ketoglutaric acid to generate oxaloacetic and pyruvic acids, respectively, which are important components of the citric acid cycle. 

Both aminotransferases are highly concentrated in the liver. AST is also diffusely represented in the heart, skeletal muscle, kidneys, brain and red blood cells. ALT is present only at low concentrations in skeletal muscle and kidney. An increase in ALT serum levels is, therefore, a more specific indicator for liver damage. In the liver, ALT is localized solely in the cellular cytoplasm, whereas AST is 20% cytosolic and 80% mitochondrial [28]. Preclinical assays currently include some combination of plasma AST, ALT, alkaline phosphatase (ALP) and lactate dehydrogenase (LDH) activities in addition to bilirrubin and albumin levels. Histological analyses of the hepatic tissue are also commonly used to evaluate the hepatoprotective action of extracts and isolated compounds. Among these assays, some are more specific and/or sensitive than others for detecting liver toxicity [29]. 

The involvement of free radicals in the pathogenesis of liver injury has been investigated for many years [30]. Various experimental studies found that toxins and drugs cause accumulation of reactive oxygen species (ROS) like superoxide, hydroxyl radical, and hydrogen peroxide in hepatocytes. This accumulation of ROS is the main cause of oxidative stress, an imbalance between increased exposure to free radicals and antioxidant defenses. The latter comprise both small molecular weight antioxidants, such as glutathione, and antioxidant enzymes. Antioxidant enzymes are catalase (CAT), superoxide dismutase (SOD), glutathione reductase (GR) and glutathione peroxidase (GPx) (Figure 1). Free radicals cause direct damage to critical biomolecules including DNA, lipids, and proteins hence injuring hepatocytes [31]. When ROS degrade polyunsaturated lipids, an elevation of cellular malondialdehyde (MDA) occurs. The production of this aldehyde, generally quantified as thiobarbituric acid reactive substances (TBARS) is frequently used as a biomarker to measure lipid peroxidation and the level of oxidative stress in an organism [32].

The presence of oxidative stress may be tested in one of three ways: (1) direct measurement of ROS; (2) measurement of the degree of damage to biomolecules; and (3) quantification of natural antioxidant molecules. The direct measurement of ROS might seem the preferred method, but many reactive oxygen species are extremely unstable and difficult to measure directly. Because of this, many investigators prefer to measure the damage on proteins, DNA, RNA, lipids, or other biomolecules. Although this is an indirect approach, many markers of damage are considerably stable and therefore provide a more reliable method to measure oxidative stress. Another approach is to measure the levels of antioxidant enzymes and other redox molecules which can counterbalance the ROS generated in the cell. Assays are available to measure the activity of specific antioxidant enzymes, such as catalase and superoxide dismutase. Additionally, there are assays that can test the antioxidant capacity of certain biomolecules and food extracts.

## 3. Main Hepatotoxic Agents used in Hepatoprotective Studies

Toxins and drugs are among the basic etiopathogenetic agents of acute liver failure in Western countries [33]. Nevertheless, chemical toxins (including acetaminophen, carbon tetrachloride, thioacetamide, ethanol, d-galactosamine, diethylnitrosamine and dimethylnitrosamine) are often used as the model substances causing experimental hepatocyte injury under both *in vivo* and *in vitro* conditions [10,34,35,36,37,38,39,40,41,42,43,44,45,46,47].

In the liver, CCl_4_ is metabolically activated by cytochrome P450-dependent mixed oxidases in the endoplasmic reticulum to form the CCl_3_ radical that combines with cellular lipids and proteins in the presence of oxygen to induce lipid peroxidation by hydrogen abstraction [48,49]. This results in structural changes in the endoplasmic reticulum and other membranes and losses in metabolic enzyme activations with the consequent impairment of liver functions. 

Ethanol, a fat-soluble non electrolyte, which is readily absorbed, diffuses rapidly into the circulation and distributes uniformly throughout the body. Ethanol is almost exclusively metabolized in the body by enzyme-catalyzed oxidative processes. The acetaldehyde formed in the first step is further oxidized to acetate, which is then converted into carbon dioxide via the citric acid cycle. Ethanol or its metabolites can also cause auto-oxidation of the hepatic cells either by acting as a pro-oxidant or by reducing the antioxidant levels resulting in marked hepatotoxicity [50]. Lipid peroxidation and associated membrane damage is a key feature of alcoholic liver injury, causing liver fibrosis leading to the development of irreversible cirrhosis. 

Paracetamol or acetaminophen is a widely used over the counter analgesic (pain reliever) and antipyretic (fever reducer). However, paracetamol overdosing causes severe hepatotoxicity that leads to liver failure in both humans and experimental animals [51,52,53]. At therapeutic doses acetaminophen is rapidly metabolized in the liver principally through glucuronidation and sulfation reactions and only a small portion is oxidized by cytochrome P-450 into highly reactive and cytotoxic intermediates. These molecules, N-acetyl-p-benzoquinone-imine (NAPQI) or N-acetyl-*p*-benzosemiquinone imine (NAPSQI) [54], can be quickly conjugated to hepatic glutathione [55,56]. Both NAPQI and NAPSQI are chemically very active and their structures indicate that they are capable of taking part in free radical reactions. Consequently, paracetamol overdosing can lead to a number of unfavorable consequences, especially those affecting the liver [57]. A large dose of the drug causes depletion of the hepatic cellular glutathione (GSH). NAPQI reacts rapidly with GSH, a phenomenon that exacerbates oxidative stress in conjunction with mitochondrial dysfunction. The GSH depletion, occurring in acute hepatotoxicity, gives free way to the highly reactive intermediates, whose actions on structural and functional molecules affects the liver functions and leads to massive hepatocyte necrosis, liver failure or death [58,59,60]. Since oxidative stress and GSH depletion contributes to the acetaminophen induced liver injury, the agents with antioxidant properties and/or GSH preserving ability may provide a preventive action against hepatocellular injury [61]. 

*N*-Galactosamine (d-GalN) is one of the most useful experimental hepatotoxins for screening and investigating hepatoprotective drugs. The hepatotoxicity of d-GalN is attributed to its metabolism in the liver, which causes a decrease in several uracil nucleotides. As a result, it inhibits RNA and protein synthesis and disturbs the biosynthesis of glycoproteins what deteriorates the cellular membranes [62,63]. The latter, in turn, disturbs the calcium homeostasis and the mitochondrial respiration [64] and leads to an excessive generation of ROS. One of the most important antioxidant enzymes in hepatocytes, SOD, is exhausted and the accumulation of ROS aggravates the damage in hepatocytes and mitochondria, which will result in the leak of AST and ALT [65,66]. 

Thioacetamide, a selective hepatotoxin is well known as an inducer of hepatic failure within a short period of time after the administration of the drug [67]. It undergoes extensive metabolism to acetamide and thioacetamide S-dioxide by the mixed function oxidase system [68]. The thioacetamide S-dioxide is a highly reactive compound [69,70]. Its binding to tissue macromolecules induces hepatic necrosis [70].

## 4. Obtainment of Fungi Extracts: Basidioma or Ascoma *versus* Mycelia Biomass

**Figure 2:**
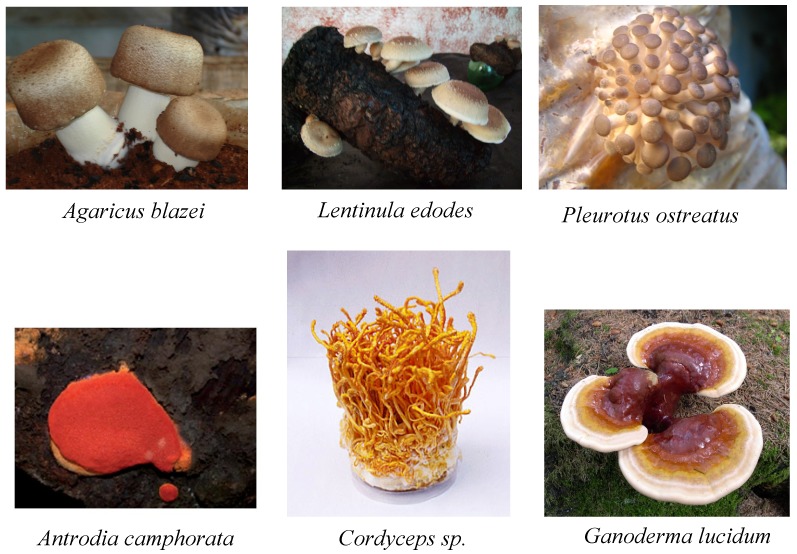
Mushrooms (Ascomycota and Basidiomycota) used in hepatoprotective studies.

Edible and medicinal mushrooms can be cultivated using several methods. Some methods are extremely simple and rustic and demand low technology. To this category belong the commercial cultures whose purpose is to obtain the basidiomata or ascomata (Figure 2). The growth of an edible mushroom, however, is a lengthy and complex process involving the use of solid composts or lignocellulosic beds, such as straw or cotton, and a long cultivation period. In addition to dried mushrooms, alternative or substitute mushroom products are their mycelia, mainly derived from submerged cultures. Growing mushroom mycelia in liquid cultures with a defined nutrient content has long been a simple and fast alternative method to produce fungal biomass [71]. These mycelia can be used as food and food-flavoring material, or in the formulation of nutraceuticals and functional foods. Modern submerged fermentation practices possess advantages such as elevated growth rates with a consequent reduction in the time for bioactives production and the possibility of optimizing the culture medium composition and the physicochemical conditions. Since the conditions of cultivation must be strictly controlled (pH, temperature, aeration, *etc.*), the production of bioactives of interest becomes more consistent and reproductive. For using the mycelial biomasses, it is necessary to prove that they are similar to the mushroom. Some studies have already shown that the mycelial biomasses of different medicinal fungi possess pharmacologic properties comparable to those of the mushroom [72,73,74,75,76,77]. Around 15% of the bioactives are presently derived from mycelial extracts of mushrooms [11], but this fraction tends to increase considering the positive results that were obtained by several research groups and the great number of investigators working in this field. 

## 5. Hepatoprotective Effects of Mushroom Extracts

Several studies have demonstrated the protective effects of herbals against experimentally induced liver injury. Additionally, a number of herbals show promising activity including silymarin against liver cirrhosis, *Phyllantus amarus* against chronic hepatitis B, glycyrrhizin for treating chronic viral hepatitis, and some herbal combinations from China and Japan that have been scientifically proven to be effective for treating liver diseases [78]. Silymarin, a reference drug, is a mixture of flavonoids and polyphenols. Silymarin has membrane-stabilizing and antioxidant activities, it promotes hepatocyte regeneration, reduces inflammatory reactions, and inhibits fibrogenesis. These properties have been established in experimental and clinical trials [79]. The compounds that are responsible for the medicinal properties of herbals are usually secondary metabolites.

The particular characteristics of growth and development of mushrooms in nature result in an accumulation of a great variety of secondary metabolites such as phenolic compounds, poly-saccharides, terpenes and steroids, several of them with biological activities. All these activities contribute in giving to the mushrooms a great potential as generators of bioactive compounds useful for promoting human health. This includes not only their consumption as foods but also their role as producers of biomolecules with specific pharmacological properties in laboratory cultures under strictly controlled conditions [9,11].

Actions of edible and medicinal mushroom bioactives on the hepatic functions have been investigated mainly by feeding animals with the whole mushroom of with non-purified extracts or by incubating liver tissue preparations with total or semi-purified extracts. Hydroalcoholic, alcoholic and aqueous extracts from both the fungal basidiomata and the mycelia have been evaluated. 

Aqueous extracts of *Volvariella volvacea*, *Lentinula edodes*, *Flammulina velutipes*, *Auricularia auricular*, *Tremella fuciformis*, *Grifola frondosa* and *Tricholoma lobayense* were screened for their hepatoprotective activities using paracetamol-induced liver injury (doses of 1.0 g/kg body weight) in rats as the model of chemical hepatitis. The results of enzymatic assays showed that paracetamol caused an acute hepatic toxicity resulting in a striking elevation of the levels of serum transaminases (AST and ALT). The extracts from the basidiomas of *L. edodes* and *G. frondosa* (100 mg/kg body weight) exerted a highly significant hepatoprotective effect by reducing the paracetamol-induced acute elevation of the AST and ALT levels, while the mycelial aqueous extract of *T. lobayense* exhibited hepatoprotective activity only at the higher dose of 300 mg/kg body weight. The extracts of the other four mushrooms did not lower the serum levels of these transaminases. As possible mechanisms for the hepatoprotective effects of the *L. edodes*, *G. frondosa* and *T. lobayense* extracts, several possibilities have been suggested: (1) preservation of the structural integrity of the hepatocytes membranes; (2) prevention of the fall of the GSH levels by acting on enzymes involved in the GSH redox cycle; (3) scavenging of the free-radicals originated from the paracetamol metabolism, which could be brought about by the antioxidant compounds of the mushroom extracts [22].

The results of representative studies about hepatoprotective effects of several mushroom crude extracts are summarized in Table 1 together with an appraisal of the main contribution of each study. The taxonomic position of the species mentioned here can be inferred from Table 2. From the 28 species listed in Table 1 only two belong to Ascomycota. Among the Basidiomycota species, the majority belongs to two orders, Polyporales and Agaricales. 

**Table 1:** The most frequently used mushrooms in hepatoprotection studies and the main contribution of each study.

Mushroom	Hepatic damage inducing drug	Main contribution	Ref.
*Antrodia camphorata* *Armillariella tabescens*	Ethanol CCl_4_	Aqueous extracts reduce the levels of AST, ALT, ALP and bilirubin.	[80,81,82]
*Lentinula edodes*	Galactosamine Dimethyl-nitrosamine	Hot water and ethanol extracts of mushrooms reduce the levels of the classical markers of hepatic damage. The hepatoprotective effect of the hot water mycelial *L. edodes* extract was attributed to the presence of two phenolics, syringic acid and vanillic acid.	[37,83,84,85]
*Macrocybe gigantea*	CCl_4_	It was suggested that the hepatoprotection could be conferred by the presence of high amounts of antioxidant phenolics and flavonoids.	[47]
*Pleurotus ostreatus*	CCl_4_ Ethanol	Aqueous extracts of *P. ostreatus* contains high concentrations of cysteine, methionine and aspartic acid.The extracts improve the antioxidant status and revert the hepatic damage.	[3,23,85 ,86]
*Pleurotus florida*	Thioacetamide Paracetamol	Hepatic protection evaluated by monitoring the plasma levels of AST, ALT, ALP, bilirubin, cholesterol and proteins.	[44,87]
*Pleurotus cornucopiae*	CCl_4_	The hepatic protection was confirmed by histological and electromicroscopical findings. Main components in the extract are D-β-(1→3)-glucans, ergosterol, mannitol, phenolic compounds, linoleic acid, peptides and other carbohydrates.	[88,89]
*Morchella esculenta*	CCl_4_ Ethanol	The hepatoprotective effect is supported by biochemical determinations and histopathological observations.	[90]
*Cordyceps TCM-700C*	CCl_4_	The extract significantly enhances the anti-oxidative constituent GSH and increases the activities of antioxidant enzymes such as catalase, GPx, GR and SOD.	[45]
*Agaricus blazei*	Diethyl-nitrosamine CCl_4_	The hepatocyte replication rate was estimated by the index of the proliferating cell nuclear antigen (PCNA) positive hepatocytes and the appearance of putative preneoplastic hepatocytes through the expression of the placental form of the enzyme glutathione S-transferase (GST-P). The PCNA labeling index, and the number of GST-P positive hepatocytes are lower in rats that received previous *A. blazei* treatment. Active components in the aqueous extract of *A. blazei* could be acting directly or indirectly on the hepatic cell membrane.	[36,91,92]
*Antrodia cinnamomea*	Ethanol	Mycelia rich in polyssccharides and triterpenoids were produced in large-scale fermentation. The cytotoxicity and the apoptosis-associated phosphatidyl serine redistribution of plasma membrane induced by ethanol are effectively reduced by 500 mg/L extract.	[93]
*Panus giganteus*	Thioacetamide	The aqueous extract restores the levels of serum liver biomarkers (ALP, ALT, AST, bilirubin, albumin, total protein) and oxidative stress parameters (TBARS) to values comparable to those of the treatment with the standard drug silymarin.	[10]
*Calocybe indica*	CCl_4_	The ethanolic extract restores the liver antioxidant status.	[46]
*Phellinus rimosus*	CCl_4_	The amelioration of liver toxicity by the ethyl acetate extract was evident from its significant effect on the levels of serum ALT, AST and ALP. The results suggest that the hepatoprotective effect of *P. rimosus* is possibly related to its free radical scavenging activity.	[94]
*Astraeus hygrometricus*	CCl_4_	Reduction of the levels of the classical markers of hepatic damage; the hepatic antioxidant status is restored.	[95]
*Coprinus comatus*	Alloxane CCl_4_	The extract showed anti-oxidant potential, and the hepato-protection was observed in liver cross sections.	[96]
*Funalia trogii*	Deltametrin	Associated with vitamin E the extract prepared in cold buffer reduces the hepatic damage evaluated by the diminution of the classical markers; the treatment restores the antioxidant status.	[97]
*Ganoderma lucidum*	CCl_4_ Benzo[a]-pyreneEthanol	The extracts have potent antioxidant and radical-scavenging effects, which contribute to hepato-protection; aqueous and alcoholic extracts exert protective actions against acute hepatitis; the extracts present free-radical scavenging ability. The hot water extract is also able to protect against renal injury; these effects were attributed to the inhibitory activities of the extract on the membrane lipid peroxidation and free radical formation, or to the free radical scavenging ability.	[35,38,98,99,100,101]
*Ganoderma tsugae*	CCl_4_	Extracts present anti-fibrotic actions and diminish the levels of ALT and AST; the extract significantly decreases the prothrombine time.	[102]

**Table 2:** Taxonomic position of the species mentioned and species fungorum current names [103].

PHYLUM	CLASS	ORDER	FAMILY	SPECIES CITED	SPECIES FUNGORUM CURRENT NAME
Ascomycota	Sordariomycetes	Hypocreales	Cordycipitaceae	*Cordyceps*	*Cordyceps* Fr*.*
	Pezizomycetes	Pezizales	Morchellaceae	*Morchella esculenta*	*Morchella esculenta* (L.) Pers
Basidiomycota	Tremellomycetes	Tremellales	Tremellaceae	*Tremella fuciformis*	*Tremella fuciformis* Berk.
	Agaricomycetes	Auriculariales	Auriculariaceae	*Auricularia auricular*	*Auricularia auricula-judae* (Bull.) Quél.
		Cantharellales	Hydnaceae	*Hericium erinaceus*	*Hericium erinaceus* (Bull.) Pers.
		Hymenochaetales	Hymenochaetaceae	*Phellinus rimosus*	*Phellinus rimosus* (Berk.) Pilát
		Polyporales	Fomitopsidaceae	*Antrodia camphorata* *Antrodia cinnamomea*	*Taiwanofungus camphoratus* (M. Zang & C.H. Su) Sheng H. Wu, Z.H. Yu, Y.C. Dai & C.H. Su *Antrodia cinnamomea* T. Chang & W.N. Chou
			Meripilaceae	*Grifola frondosa*	*Grifola frondosa* (Dicks.) Gray
			Polyporaceae	*Panus giganteus* *Funalia trogii*	*Panus giganteus* (Berk.) Corner *Trametes trogii* Berk
			Ganodermataceae	*Ganoderma lucidum* *Ganoderma tsugae*	*Ganoderma lucidum* (Curtis) P. Karst. *Ganoderma tsugae* Murrill
		Boletales	Diplocystidiaceae	*Astraeus hygrometricus*	*Astraeus hygrometricus* (Pers.) Morgan
		Agaricales	Pluteaceae	*Volvariella volvacea*	*Volvariella volvacea* (Bull.) Singer
			Pleurotaceae	*Pleurotus ostreatus* *Pleurotus florida* *Pleurotus cornucopiae* *Pleurotus eryngii*	*Pleurotus ostreatus* Jacq.) P. Kumm *Pleurotus floridanus* Singer *Pleurotus cornucopiae* (Paulet) Rolland *Pleurotus eryngii* (DC.) Quél.
			Physalacriaceae	*Flammulina velutipes* *Armillariella tabescens*	*Flammulina velutipes* (Curtis) Singer *Armillaria tabescens* (Scop.) Emel*e*
			Marasmiaceae	*Lentinula edodes*	*Lentinula edodes* (Berk.) Pegler
			Tricholomataceae	*Tricholoma lobayense* *Macrocybe gigantea*	*Macrocybe lobayensis* (R. Heim) Pegler & Lodge *Macrocybe gigantea* (Massee) Pegler & Lodge
			Lyophyllaceae	*Calocybe indica*	*Calocybe indica* Purkay. & A. Chandra
			Agaricaceae	*Agaricus blazei* *Coprinus comatus*	*Agaricus blazei* Murrill *Coprinus comatus* (O.F. Müll.) Pers
			Strophariaceae	*Pholiota dinghuensis*	*Pholiota dinghuensis* Z.S. Bi

The scientific names used by the various authors were preserved, but the species fungorum current names were also listed in Table 2. With a few exceptions, the species listed in Table 2 have also been enrolled as producing some type of biological effect. It should be noted that several models of hepatic injury were used so that caution must be taken when interpreting the results. 

## 6. Molecular Species Responsible for the Hepatoprotective Effects of the Fungi

It is obviously of interest to identify the molecular species responsible for the hepatoprotective effects. In this respect, unfortunately, research is still incipient. A few studies, however, have been conducted with purified or semi-purified molecules. In the following text only those studies are mentioned in which purified molecules were used or a strong indirect evidence has been presented about the participation of a specific molecule in the hepatoprotective effect.

### 6.1. Anthraquinol from Antrodia Cinnamomea Ethanolic Mycelial Extract

**Figure 3:**
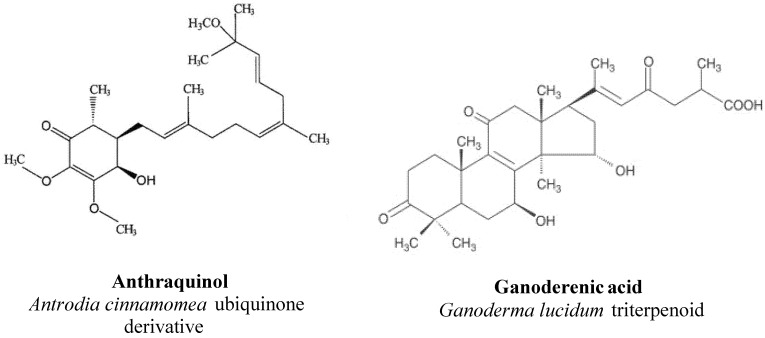
Two representative molecules obtained from mushrooms with hepatoprotective properties.

*Antrodia cinnamomea* has received considerable attention with regard to its possible health benefits, especially for its hepatoprotective effects against various drug-, toxin-, and alcohol-induced liver diseases. Purified anthraquinol, a ubiquinone derivative was isolated from the basidioma and the mycelium of *Antrodia cinnamomea* (Figure 3). 

Pretreatment with the compound significantly inhibited the ethanol-induced AST, ALT, ROS, NO, MDA productions and GSH depletion in human hepatoma cell lines (HepG2 cells). Western blot and quantitative real time PCR analysis showed that anthroquinonol enhanced the Nrf-2 activation and its downstream antioxidant gene HO-1 via the mitogen-activated protein kinases (MAPK) pathway. This mechanism was then confirmed in vivo in an acute ethanol intoxicated mouse model: elevation of serum ALT and AST levels, hepatocellular lipid peroxidation and GSH depletion were prevented by an ethanolic *A. cinnamomea* extract in a dose-dependent manner. The extract significantly enhanced HO-1 and Nrf-2 activation via MAPKs, what is consistent with the in vitro studies. The ethanol-induced hepatic swelling and hydropic degeneration of hepatocytes was significantly inhibited by the extract in a dose-dependent manner. These results provide a scientific basis for the hepatoprotective effects of *A. cinnamomea* and also imply that anthraquinol, a potent bioactive compound may be responsible, at least in part, for the hepatoprotective activity of *A. cinnamomea* [104].

### 6.2. Triterpenoids, Polysaccharides and Peptides from *Ganoderma lucidum*

The most studied mushroom with respect to hepatoprotective effects is *Ganoderma lucidum.* This fact is not surprising because *G. lucidum* is, indubitably, the most studied medicinal mushroom. Approximately 400 chemical substances have been isolated from *G. lucidum*, which include mainly polysaccharides, triterpenoids, nucleosides, ergosterols, fatty acids, proteins/peptides, and trace elements. Particularly polysaccharide and triterpenoid components in *G. lucidum* have been proposed as the bioactive constituents responsible for the protective activities against toxin-induced liver injury [105,106,107]. In a broad review about the hepatoprotective properties of *G. lucidum*, Gao *et al*. [106] collected evidence to suggest possible molecular mechanisms to explain its hepatoprotective actions. Among these mechanisms, the authors include antioxidant and radical-scavenging activity, modulation of hepatic Phase I and II enzymes, inhibition of β-glucuronidase, antifibrotic and antiviral activity, modulation of nitric oxide production, maintenance of hepatocellular calcium homeostasis, and immunomodulatory effects. 

Data from *in vitro* and animal studies indicate that *G. lucidum* extracts, mainly polysaccharides or triterpenoids, exhibit protective activities against liver injury induced by toxic chemicals (e.g., CCl_4_) and *Bacillus* Calmette-Guerin (BCG) plus lipopolysaccharide (LPS). The fungus also showed antihepatitis B-virus (HBV) activity in a duckling study. Recently, a randomized placebo-controlled clinical study showed that treatment with *G. lucidum* polysaccharides for 12 weeks reduced hepatitis B e antigen (HBeAg) and HBV DNA by 25% (13/52) in patients with HBV infection. The mechanisms of the hepatoprotective effects are still undefined. Evidence suggests that antioxidant and radical scavenging activity, modulation of hepatic phase I and II enzymes, inhibition of b-glucuronidase, antifibrotic and antiviral activity, modulation of nitric oxide production, maintenance of hepatocellular calcium homeostasis, and immunomodulatory effects might be involved [107]. 

The effects of total triterpenoids extracts from *G. lucidum* on two different experimental liver injury models induced by carbon tetrachloride and d-galactosamine were extensively studied in mice [35,40,108,109]. Administration of the extract (80 mg/kg) significantly inhibited the increase of serum ALT and liver triglyceride levels in the models, effects similar to those of malotilate, a known reference substance for this kind of protective effects [110]. The *G. lucidum* extract also antagonized the decrease of the SOD activity and the GSH content and inhibited the increase of the MDA content in the carbon tetrachloride and d-galactosamine liver-injured mice. It could equally improve the histopathological changes. These observations are likely to indicate that triterpenoids isolated from *G. lucidum* have a powerful protective effect against liver damage induced by carbon tetrachloride and d-galactosamine. Their hepatoprotective effects were perhaps related to the ability to increase the activity of free radical scavenging enzymes and, thus, to raise the ability of antioxidation. It should be stressed that ganoderenic acid (Figure 2), one of the triterpenoids found in *G. lucidum*, was proven to be a potent inhibitor of β-glucuronidase activity, an indicator of hepatic damage [111]. 

The hepatoprotective activity of peptides from *Ganoderma lucidum* was evaluated against d-galactosamine (d-GalN)-induced hepatic injury in mice. *G. lucidum* peptides were administered via gavage daily for two weeks at doses of 60, 120 and 180 mg/kg, respectively. The d-GalN-induced hepatic damage was manifested by a significant increase in the activities of marker enzymes (AST, ALT) in serum, by the increased MDA levels in the liver, and by significant decreases in the activity of SOD and in the GSH level in the liver. Pretreatment of mice with *G. lucidum* peptides maintained these parameters at their normal values. These biochemical results were supplemented by histo-pathological examination of liver sections. The best hepatoprotective effects of the *G. lucidum* peptides were observed after treatment with the dose of 180 mg/kg as deduced from the biochemical parameters and liver histopathological examinations. Results of this study revealed that the *G. lucidum* peptides can produce a significant diminution of the d-GalN-induced hepatocellular injury [40]. 

### 6.3. Polyssacharide from Pleurotus Ostreatus Mycelium

Insoluble non-starch polysaccharides from the *P. ostreatus* mycelium were evaluated as pre-treatment to prevent the carbon tetrachloride induced hepatic damage in rats. The polysaccharides (100 and 200 mg/kg) were administered orally each day for 15 days before carbon tetrachloride (1.5 mL/kg). The effect of the polysaccharides treatment was also examined in normal rats. Normal groups treated with the polysaccharides showed significant decreases in serum activities of the liver enzymes, lipid peroxides and nitric oxide in the liver. The GSH and total proteins contents of the liver homogenate also increased after treatment with polysaccharides for 15 days. In carbon tetrachloride-treated rats, significant elevation of the serum liver enzymes, increased lipid peroxides and nitric oxide in the liver, and depletion of hepatic GSH were observed. Pretreatment with the polysaccharides significantly ameliorated the tested parameters when compared with the carbon tetrachloride-treated group. Histopathological examination of the hepatic tissue revealed that polysaccharide administration alone protected hepatocytes from the damage induced by carbon tetrachloride [112]. Similar results were obtained in a study in which hot-water polysaccharopeptides from the culture broth of the *P. ostreatus* mycelium were tested against injury induced by thioacetamide in mice [113].

### 6.4. Mycelial Polysaccharide from Pholiota Dinghuensis

The production of a mycelial polysaccharide from *Pholiota dinghuensis* Bi was optimized. Biochemical assays and histopathological analyses showed that the crude mycelial polysaccharide exerted a significant hepatoprotective action in a dose-dependent manner against carbon tetrachloride-induced acute liver injury in mice. The polysaccharide prevented the increased activities of serum ALT and AST, reduced the formation of malondialdehyde and enhanced the activities of superoxide dismutase and glutathione peroxidase [114].

### 6.5. Water-Soluble Polysaccharide of Pleurotus Eryngii

An aqueous extract of *P. eryngii*, rich in polysaccharide, was investigated for its actions on an *in vivo* mouse model of liver injury induced by carbon tetrachloride. The extract significantly increased the activities of antioxidant enzymes and effectively removed the free radicals in the injured liver. Furthermore, in a high-fat-load mouse model, the polysaccharide rich extract not only remarkably decreased the levels of total cholesterol, total triglycerides, and low-density lipoprotein cholesterol, but also produced an increase in the high-density lipoprotein cholesterol levels. Histopathological observations indicated that the polysaccharide rich extract could effectively prevent excessive lipid formation in the liver tissue. The authors suggested that the polysaccharide rich extract of *P. eryngii* can be used as a valuable functional food additive for hypolipidemic and hepatoprotective treatments [115].

### 6.6. Mycelial Endo-Polysaccharides of Hericium Erinaceus

Three fractions of endo-polysaccharides from the mycelium of *H. erinaceus,* grown on tofu whey, were obtained by fractional precipitation with an ethanol gradient. The studies for evaluating the antioxidant potential and the hepatoprotective effects against carbon tetrachloride injury revealed that each of the polysaccharides had different activities in several evaluation systems. The polysaccharide precipitated with 80% ethanol had the strongest antioxidant activity *in vitro* and a potent hepatoprotective effect *in vivo*. The authors suggest that the hepatoprotective effect may be due to its potent antioxidant capacity. According to the authors the *H. erinaceus* polysaccharides could be exploited as antioxidant products and as supplements in the prevention of hepatic diseases [116].

### 6.7. Lectin from Pleurotus Florida

A lectin from *P. florida* was investigated for its actions in reverting the arsenic-induced hepatic damage in rats. The authors observed significant alterations in the levels of antioxidant enzymes, oxidative stress intermediates and SOD_2_ gene expression profile on arsenic exposure and these alterations were restored by co-administration of the *P. florida* lectin which was as potent as the standard antioxidant viz. ascorbic acid [117]. 

## 7. Conclusions

It can be concluded from the quite numerous and generally consistent reports that were detailed above that many mushroom extracts possess hepatoprotective properties against liver injury caused by toxic chemicals. In principle at least, scientific evidence seems, thus, to validate the use of mushrooms in folk medicine. The mushrooms may represent a new alternative to the limited therapeutic options that exist presently in the treatment of liver diseases or their symptoms, and they should be considered as such in future studies. In general terms it seems that phenolics, triterpenes, polysaccharides and peptides are the main classes of compounds which could be responsible for the hepatoprotective activity of the mushroom extracts. Unfortunately, precise identifications of specific molecules involved in the hepaprotective effect are scarce. This is an area still demanding considerable efforts. The potent hepatoprotective activities of the chemically defined molecules isolated from natural materials may represent and exciting advance in the search for effective liver protective agents, especially now when there is an urgent need for new innovative drugs. Further studies including clinical trials need to be carried out to ascertain the safety of these compounds as good alternatives to conventional drugs in the treatment of liver diseases. Another perspective results from a recent study in which hepatoprotection by an *Agaricus blazei* extract on the paracetamol-induced injury was investigated [118]. It was found that the extract was quite effective in normalizing several of the classical markers of liver injury such as the release of enzymes into the plasma and the lipid peroxidation levels. It failed, however, in restoring the gluconeogenic activity of the liver. Glucose synthesis is a very important function of the liver and one would expect it to be fully restored by the protective agents. It would thus be highly desirable to incorporate functional parameters, such as gluconeogenesis, into the experimental protocols of studies aiming to attribute or refute hepatoprotective actions.
